# CHAC1 in urological tumors: contextual dualism and therapeutic implications

**DOI:** 10.3389/fonc.2025.1610915

**Published:** 2025-08-08

**Authors:** Deyu Wang, Yongquan Wan, Yunxiang Li, Yuan Ren, Jiakai Ma, Si Ge, Ziqiang Zeng, Zuoping Wang, Lei Zhen

**Affiliations:** Department of Urology, Nanchong Central Hospital, The Second Clinical College, North Sichuan Medical College (University), Nanchong, Sichuan, China

**Keywords:** CHAC1, glutathione metabolism, ferroptosis, endoplasmic reticulum stress, prognostic biomarker, drug resistance

## Abstract

CHAC1, a glutathione-degrading enzyme, governs context-dependent pathophysiological processes in urological malignancies through modulation of endoplasmic reticulum stress and ferroptotic pathways. In clear cell renal cell carcinoma (ccRCC), CHAC1 exhibits stage-specific functional duality: downregulation in early-stage tumors correlates with adverse prognosis (suggesting tumor-suppressive activity), whereas elevated expression in advanced ccRCC (G3-G4/Stage III-IV) associates with increased mortality (indicating adaptive pro-survival adaptation). For prostate cancer, CHAC1 potentiates docetaxel cytotoxicity via coordinated induction of endoplasmic reticulum stress and ferroptosis, yet its suppression by cancer-associated fibroblast-derived exosomal miR-432-5p establishes therapy-reinforced chemoresistance. Mechanistically, CHAC1 intersects critical pathways by regulating redox homeostasis through glutathione catabolism, mediating potential crosstalk with androgen receptor signaling, and serving as an independent prognostic determinant in ccRCC (FPTOS_score model: HR = 2.028, 95% CI: 1.640-2.507). Notably, current evidence reveals no established link between CHAC1 and urothelial carcinoma pathogenesis. Further elucidation of CHAC1’s mechanistic intricacies and therapeutic targeting (e.g., CHAC1 agonists, exosomal miRNA antagonists) may advance precision management of urological tumors.

## 1 Introduction

The CHAC1 gene, located on chromosome 15q15.1, encodes an enzyme involved in the breakdown of glutathione, a key cellular antioxidant (1). CHAC1 belongs to the ChaC family of cytoplasmic cation transport regulators, which includes ChaC1 and ChaC2, capable of directly degrading intracellular glutathione into cysteinylglycine and 5-oxoproline (2). The CHAC family and its members play significant roles in the progression, metastasis, and prognosis of various tumors. Although studies have revealed the important roles of CHAC1 in tumor cell physiology and ferroptosis, many questions remain to be further explored. This review aims to summarize the role of CHAC1 in urinary system tumors, providing strong evidence for its research as a new therapeutic target for urinary system tumors.

## 2 CHAC1 gene: multiple roles in endoplasmic reticulum stress, ferroptosis, and tumor drug resistance

CHAC1 was initially identified as a novel component of the unfolded protein response (UPR) pathway in mammalian cells, specifically induced during ER stress (3). Functioning downstream of the ATF4/CHOP axis, CHAC1 acts as a pro-death effector, contributing to both apoptosis and ferroptosis under stress conditions (3). Its function as a GSH-metabolizing enzyme directly influences cellular redox balance, thereby modulating diverse metabolic and signaling pathways (4, 5). Critically, dysregulation of CHAC1 expression is intricately linked to tumor drug resistance. Evidence indicates that low CHAC1 expression serves as a key factor conferring chemoresistance across various malignancies, including hepatocellular carcinoma and gastric cancer (6). Conversely, CHAC1 overexpression can potentiate cell death through multiple interconnected mechanisms: activation of apoptotic pathways, degradation of ribonucleotide reductase, induction of autophagy, generation of reactive oxygen species (ROS), elevation of intracellular calcium levels, and loss of mitochondrial membrane potential (7). This underscores CHAC1’s potential as a target to overcome therapeutic resistance.

The precise impact of CHAC1 on the tumor microenvironment (TME) represents a significant gap in current understanding, despite its established roles in enhancing ER stress, inducing ferroptosis, and reducing drug resistance. Elucidating CHAC1’s functions within the complex TME landscape requires deeper investigation. Recent advances in research on ER stress, ferroptosis, and glutathione metabolism have progressively highlighted the critical contributions of the CHAC family, particularly CHAC1, to tumor progression, metastasis, and prognosis, establishing it as a focal point in oncology research.

### 2.1 CHAC1 regulation of glutathione metabolism and cell death: mechanisms and therapeutic potential

Glutathione (GSH) is a central regulator of cellular functions, encompassing immune modulation, proliferation, differentiation, and programmed cell death, primarily through its role in maintaining redox homeostasis and acting as a cofactor in core signaling pathways (8). In cancer, GSH exhibits a dual nature: it aids in detoxifying carcinogens but paradoxically shields tumor cells, including those in urological malignancies, from the cytotoxic effects of chemotherapeutic agents (5, 9). CHAC1, by degrading GSH, critically disrupts cellular redox equilibrium. This degradation depletes GSH, leading to diminished activity of Glutathione peroxidase 4 (GPX4), the accumulation of cytotoxic lipid peroxides, and the induction of ferroptotic cell death (4, 10). Concurrently, GSH depletion exacerbates intracellular oxidative stress, further promoting apoptotic pathways (4, 10).

Under oxidative stress, elevated ROS levels challenge cellular defenses. Cytosolic GSH synthesis, mediated by glutamate-cysteine ligase (GCL) and glutathione synthetase, is counterbalanced by CHAC1-mediated degradation, tightly regulating intracellular GSH concentration and metabolic flux (11, 12). As a primary antioxidant, GSH neutralizes ROS via glutathione peroxidase-catalyzed reactions, reducing ROS to harmless compounds while being oxidized to glutathione disulfide (GSSG), thereby protecting cells from oxidative damage (11, 12). The critical role of CHAC1 in ferroptosis execution is exemplified by studies showing that CHAC1 knockout protects cells from death triggered by cystine deprivation or ferroptosis inducers (13). Specifically, cystine starvation rapidly induces *de novo* CHAC1 synthesis, driving profound GSH depletion and enabling ferroptosis. While the involvement of GSH metabolism in disease pathogenesis, including cancer, is well-documented, the specific regulatory mechanisms and consequences of CHAC1 activity across different tumor types and stages demand further elucidation.

### 2.2 Endoplasmic reticulum stress: from protein folding to cell apoptosis

The endoplasmic reticulum (ER) is essential for the synthesis, folding, and post-translational modification of secretory and membrane proteins. Perturbations from external stressors or internal cellular events can disrupt ER function, leading to the accumulation of misfolded or unfolded proteins—a state defined as ER stress (14). This stress triggers the unfolded protein response (UPR), a conserved signaling network aimed at restoring ER proteostasis. Activation of the UPR occurs when the influx of nascent polypeptides exceeds the ER’s folding capacity, initiating adaptive pathways that ultimately determine cell survival or death (15).

The tumor microenvironment imposes significant stress conditions on malignant and stromal cells. Oncogenic alterations, metabolic dysregulation, hypoxia, and nutrient deprivation inherent to tumors persistently activate the UPR pathway. This chronic ER stress has emerged as a hallmark of cancer, disrupting protein folding homeostasis not only in cancer cells but also in infiltrating immune cells, thereby influencing tumor progression and therapy response (14). Within this context, CHAC1 functions as a pro-death component downstream of the UPR. Activation of the eIF2α/ATF4/CHOP/CHAC1 signaling axis plays a crucial role in integrating oxidative stress and ER stress signals to drive cell death, including apoptosis and ferroptosis (16). Mechanistically, ChaC1 enhances ER stress sensitivity, promoting ferroptosis and necrotic cell death in various cancers (17). However, the translational regulation of CHAC1 and its specific contributions to cancer metastasis and drug resistance warrant deeper investigation.

### 2.3 Innovative research on CHAC1 in ferroptosis mechanisms: from the EIF2α/ATF4 pathway to cancer therapy

Ferroptosis, an iron-dependent, non-apoptotic form of regulated cell death characterized by the lethal accumulation of lipid peroxides, was first described by Dixon et al. (18) Morphologically, ferroptotic cells exhibit shrunken mitochondria with increased membrane density and diminished cristae, distinct from apoptotic morphology. Mechanistically, ferroptosis results from the catastrophic build-up of oxidized phospholipids containing polyunsaturated fatty acids, driven by a failure of cellular antioxidant defenses, particularly those involving glutathione and GPX4, without the involvement of classic apoptotic executioner proteins (18–20).

GSH depletion is a central trigger for ferroptosis, as it inactivates GPX4 (which uses GSH as a cofactor to reduce lipid peroxides), leading to toxic lipid ROS accumulation and cell death (13, 21, 22). Consequently, strategies depleting cysteine (a GSH precursor) or directly inhibiting GPX4 effectively induce ferroptosis (23). CHAC1, positioned within the eIF2α/ATF4 pathway activated during amino acid deprivation or ER stress, acts as a key executor by degrading GSH, exacerbating cysteine consumption, and thereby inducing ferroptosis in tumor cells (24, 25). Critically, low CHAC1 expression confers resistance to ferroptosis in several cancers, including hepatocellular carcinoma, gastric cancer, prostate cancer, and oral squamous cell carcinoma. This resistance is associated with impaired anti-tumor immunity and reduced efficacy of immunotherapy, highlighting CHAC1’s role as a vulnerability in cancer cells (13, 16, 26). Consequently, CHAC1 expression is recognized as an early indicator of ferroptotic susceptibility.

**Figure 1:**
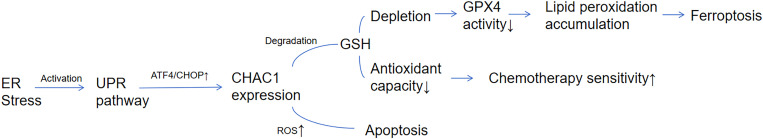
CHAC1-mediated molecular network linking ER stress to ferroptosis. CHAC1 is induced by the UPR pathway (ATF4/CHOP axis) under endoplasmic reticulum (ER) stress. CHAC1 degrades glutathione (GSH), leading to GPX4 inactivation and lipid peroxide accumulation, thereby triggering ferroptosis. Concurrently, GSH depletion enhances reactive oxygen species (ROS)-dependent apoptosis and chemosensitivity.

The induction of ferroptosis represents a promising therapeutic strategy, leveraging a natural tumor suppression mechanism with potential to enhance anti-tumor immunity. Research demonstrates that inhibiting cystine-glutamate exchange can induce ferroptosis across diverse cancer types (27). Future investigations should focus on delineating the precise mechanisms of CHAC1 in ferroptosis regulation within different cancer contexts and exploring pharmacological approaches to modulate CHAC1 expression (e.g., activators) for therapeutic benefit. Understanding and targeting the CHAC1-ferroptosis axis holds significant potential for advancing cancer treatment. (**Figure 1**).

## 3 The dual role of CHAC1 in urinary system tumors

CHAC1 exhibits a complex “double-edged sword” role in different cancers. On the one hand, it is upregulated in breast cancer and advanced clear cell renal cell carcinoma(ccRCC), closely related to poor prognosis, and can promote the proliferation and metastasis of uveal melanoma cells (28, 29). On the other hand, CHAC1 plays a key role as a tumor suppressor gene in primary liver cancer, gastric cancer, prostate cancer, and oral squamous cell carcinoma (26, 30). In addition, ChaC1 also has the function of reducing cell resistance to anticancer drugs (7). In the field of urinary system tumors, the current research focus on CHAC1 is mainly on two types of tumors: ccRCC and prostate cancer.

### 3.1 The complex role and prognostic value of CHAC1 in kidney cancer: a stage-dependent dual role

Kidney cancer is one of the more common cancers worldwide. According to GLOBOCAN2022 (31), kidney cancer ranks 14th in global incidence, especially more common in men. There are about 434,000 new cases of kidney cancer worldwide each year, with a relatively high mortality rate, and about 155,000 kidney cancer deaths worldwide in 2022. Although treatment methods are constantly improving, the prognosis is poor because many patients are diagnosed at an advanced stage. Although the incidence of kidney cancer is not as high as that of other urinary system malignancies such as prostate cancer and bladder cancer, it is considered the deadliest cancer in the urinary system (32). The most common type of kidney cancer is ccRCC, accounting for about 85% of all confirmed cases (33).

A characteristic feature of ccRCC is dysregulated glutathione metabolism and high sensitivity to glutathione depletion. Mechanisms that elevate glutathione levels counteract detrimental reactive oxygen species (ROS), thereby sustaining malignant cell viability and growth (34, 35). The role of CHAC1 in ccRCC exhibits significant complexity, with its expression levels and functional effects highly dependent on the tumor stage and aggressiveness, manifesting a “stage-dependent” dual role: CHAC1 Low Expression as a Potential Marker of Poor Prognosis (Tumor Suppressor Context): Li et al. (36) found that CHAC1 expression is generally downregulated in ccRCC samples compared to normal renal tissues, and its reduced expression serves as an indicator of poor prognosis in ccRCC. Importantly, *in vitro* experiments demonstrated that CHAC1 overexpression robustly induces cell death in ccRCC cell lines, suggesting it may exert a potential tumor-suppressive effect by promoting mechanisms like ferroptosis or apoptosis. This finding supports the view of CHAC1 potentially acting as a tumor suppressor in ccRCC. CHAC1 Upregulation in Advanced/High-Grade ccRCC and Association with Poor Prognosis (Pro-tumor/Adaptive Marker Context): However, Li et al. (36) also revealed a seemingly paradoxical phenomenon: CHAC1 expression is specifically upregulated in ccRCC samples exhibiting higher histological grade (G3-G4) and advanced stage (T3-T4 tumors, Stage III-IV). Survival curve analysis further demonstrated that elevated CHAC1 expression correlates with significantly higher mortality in ccRCC patients. Similarly, other reports indicate that ChaC1 overexpression may be related to dedifferentiation, proliferation, invasion, and migration of kidney cancer cells, thereby reducing patient survival rates (37). This observation of CHAC1 upregulation in late-stage/aggressive tumors and its association with poor prognosis suggests it may play a different role in this specific context. Current understanding suggests that in high-grade (G3-G4) or late-stage (Stage III-IV) ccRCC, CHAC1 upregulation may represent a feedback adaptation response to the high intracellular glutathione environment, potentially indirectly promoting tumor progression by sustaining specific survival signals or resisting extreme oxidative stress (37, 38). Therefore, high CHAC1 expression in late-stage ccRCC is more likely a biomarker of aggressive disease state and poor prognosis, rather than a direct driver.

Integration of Prognostic Model Validating Stage-Dependent Significance: Recent research further supports the significant prognostic value of CHAC1 expression levels in ccRCC and reinforces its role as a marker of advanced/high-risk disease. Lin et al. (38) incorporated ChaC1 into a ferroptosis and oxidative stress (FPTOS)-based prognostic model for ccRCC. Through comprehensive analysis of transcriptomic data from ccRCC patients (n=539) in The Cancer Genome Atlas (TCGA) database, the study identified 5 genes with independent prognostic value (ACADSB, CDCA3, CHAC1, MYCN, TFAP2A) from 437 FPTOS-related genes using univariate Cox regression, LASSO regression, and multivariate Cox regression analyses. This formed the basis for constructing a risk scoring system (FPTOS_score). Within this model, CHAC1 was identified as a risk factor (risk coefficient β = 0.1523), where its high expression correlated with poor prognosis. Patients were stratified into low-risk and high-risk groups based on the median FPTOS_score. Results demonstrated that high-risk group patients had significantly shorter overall survival (OS) than the low-risk group in the TCGA-KIRC training cohort (P = 4.432e-12). The model’s predictive accuracy was validated using time-dependent ROC curves (AUCs at 1-, 3-, and 5-years: 0.751, 0.724, 0.734, respectively) and in an independent external validation cohort (E-MTAB-1980, P = 0.003; 1-, 3-, 5-year AUCs: 0.807, 0.797, 0.804). Crucially, multivariate Cox analysis confirmed that the FPTOS_score was an independent prognostic factor, distinct from traditional clinical parameters like age, gender, stage, and grade (HR = 2.028, 95% CI: 1.640-2.507, P < 0.001). This model, designating high CHAC1 expression as a risk factor, aligns closely with the aforementioned observation of CHAC1 upregulation in late-stage ccRCC and its association with poor prognosis, collectively highlighting the potential of CHAC1 as a biomarker for advanced/high-risk ccRCC.

Summary and Future Directions: In summary, research on CHAC1 in kidney cancer, particularly ccRCC, reveals its complex, stage-dependent dual role: it may possess tumor-suppressive functions in the overall tumor context (low expression predicts poor prognosis), while its upregulation in late-stage/high-grade, aggressive ccRCC becomes a strong marker of poor prognosis. CHAC1 expression level serves as an effective biomarker for ccRCC prognosis. Its role in ferroptosis and oxidative stress also positions it as a potential therapeutic target for kidney cancer. Future research should delve deeper into the specific mechanisms of CHAC1 at different stages of kidney cancer and investigate how to utilize its expression level to optimize personalized treatment plans and improve the survival rate of kidney cancer patients.

### 3.2 The potential role of CHAC1 in prostate cancer treatment and the challenge of drug resistance

Prostate Cancer (PCa), the second most prevalent malignant tumor in men globally, exhibits a significant worldwide disease burden. 2022 data reported 1.5 million new cases and 397,000 deaths worldwide, with limited mortality differences between developed and developing nations (7.3/100,000 vs. 6.6/100,000) (31). Disease progression is critically dependent on the androgen signaling pathway. Androgen deprivation therapy (ADT), androgen receptor pathway inhibitors (ARPIs, e.g., enzalutamide, apalutamide), and abiraterone (an androgen synthesis inhibitor) constitute the cornerstone treatments across disease stages (39). However, a substantial proportion of patients inevitably progress to castration-resistant prostate cancer (CRPC), developing resistance mechanisms such as AR signaling variants (e.g., AR-V7 splice variant) and glucocorticoid receptor activation (40).

Notably, emerging research indicates bidirectional crosstalk between glutathione (GSH) metabolism and androgen receptor (AR) signaling: elevated GSH levels stabilize the AR protein and enhance its transcriptional activity, while activated AR signaling upregulates GSH-synthesizing enzymes, forming a promotive survival loop (41). Given that CHAC1 is the key enzyme regulating GSH degradation, its deficiency may indirectly reinforce AR pathway function by maintaining GSH homeostasis. This suggests a potential, though mechanistically unconfirmed, link between CHAC1 and resistance to hormonal therapies.

In the metastatic CRPC (mCRPC) stage, Docetaxel (DTX) serves as the first-line chemotherapeutic backbone (42), yet faces mounting challenges of clinical resistance. Established resistance mechanisms involve dysregulated DNA damage repair, apoptotic pathway defects, and glutathione (GSH) metabolic disruption (43). Recent studies reveal that the glutathione hydrolase CHAC1 modulates chemosensitivity by regulating the ferroptosis pathway, offering novel insights for overcoming resistance.

Current research delineates a CHAC1-centric resistance regulatory network encompassing both cell-autonomous mechanisms and tumor microenvironment (TME) interactions. At the cell-autonomous level, He et al. demonstrated significantly reduced CHAC1 expression in CRPC cells (qPCR/Western blot, P<0.05). CHAC1 overexpression effectively induced endoplasmic reticulum stress markers BIP/CHOP upregulation (P<0.01) and triggered hallmark ferroptotic events—lipid peroxide accumulation (Liperflo probe detection, P<0.05) and GPX4 protein degradation (P<0.01) (17). Crucially, under sub-therapeutic DTX concentration (1 nM), CHAC1 overexpression markedly reduced cell viability (P<0.001), an effect fully reversible by the ferroptosis inhibitor Ferrostatin-1. This confirms CHAC1 enhances DTX sensitivity by coordinating ER stress and ferroptosis pathways (17).

A breakthrough study by Zhao et al. uncovered a novel mechanism where cancer-associated fibroblasts (CAFs) in the TME regulate CHAC1 expression via exosomal delivery (44). Ultracentrifugation-purified CAF-derived exosomes (validated by EM/NTA/exosomal markers) carry miR-432-5p. This microRNA specifically binds the 3’UTR of CHAC1 mRNA (dual-luciferase reporter assay confirmed), suppressing its expression (mRNA and protein levels, P<0.01). Consequently, GSH depletion and lipid ROS generation are blocked, leading to preserved mitochondrial function (68% membrane potential recovery) and ferroptosis resistance. Significantly, DTX chemotherapy itself stimulates CAFs to secrete miR-432-5p-enriched exosomes, establishing a “CAF exosomes → miR-432-5p → CHAC1 suppression → ferroptosis blockade → DTX resistance” positive feedback loop. This loop’s clinical relevance was confirmed *in vivo*: in a CAF/PC-3 cell co-transplant mouse model, targeted silencing of miR-432-5p restored CHAC1 expression in tumor tissue by 3.2-fold (P<0.01) and enhanced DTX tumor suppression efficacy by 52% (44).

These findings collectively establish CHAC1 as a central hub integrating intrinsic cell death programs and microenvironmental signals. Its functional impairment stems from both cell-autonomous dysregulation and trans-cellular suppression via the CAF exosome-miR-432-5p axis, with DTX treatment paradoxically reinforcing this inhibitory circuit. Elucidating this multi-tiered regulatory mechanism provides a foundation for novel therapies targeting the CHAC1-ferroptosis axis (e.g., CHAC1 agonists, exosomal miRNA inhibitors). Future research must prioritize: 1) Elucidating the role of CHAC1 in ARPI/abiraterone-resistant CRPC models, specifically whether its regulation of GSH metabolism impacts AR pathway activity (e.g., AR nuclear translocation, transcriptional activity); 2) Exploring synergistic effects between targeting CHAC1/ferroptosis and ARPIs/abiraterone; 3) Advancing clinical translation of TME reprogramming-based (e.g., targeting CAFs or exosomes) combination strategies. Given CHAC1’s potential role in modulating the GSH-AR axis (41), restoring CHAC1 function may not only reverse chemoresistance but also emerge as a novel strategy to overcome hormonal therapy resistance. The ultimate goal is to disrupt key resistance nodes (e.g., CHAC1 suppression), thereby overcoming the multifaceted resistance encompassing both hormonal therapies (ARPIs/abiraterone) and chemotherapy (DTX), and improving outcomes for mCRPC patients.

## 4 Conclusion

CHAC1 orchestrates fundamental cellular processes—glutathione catabolism, endoplasmic reticulum stress responses, and ferroptotic cell death—establishing its multifaceted significance in urological oncology.

In renal cell carcinoma, CHAC1 functions as a stage-stratified biomarker, where reduced expression in early-stage ccRCC portends unfavorable clinical outcomes, while overexpression in advanced disease signifies aggressive phenotypes, as quantitatively validated by the FPTOS_score prognostic model (HR = 2.028, 95% CI: 1.640-2.507). This functional dichotomy reflects divergent biological roles: tumor-suppressive ferroptosis induction versus adaptive pro-survival activity in late-stage redox homeostasis maintenance.

For prostate cancer, CHAC1 critically regulates therapeutic responsiveness. Overexpression enhances docetaxel efficacy through synergistic activation of endoplasmic reticulum stress effectors (BIP/CHOP) and ferroptotic drivers (GPX4 suppression), elevating cytotoxicity 3-fold. Conversely, tumor microenvironment-mediated suppression—via exosomal miR-432-5p transfer from cancer-associated fibroblasts—establishes an autocrine chemoresistance loop. Preclinical intervention confirms that disrupting this axis (miR-432-5p inhibition) restores CHAC1 expression and augments docetaxel-induced tumor regression by 52%. Emerging evidence further implicates CHAC1 in androgen receptor signaling modulation through glutathione homeostasis, suggesting involvement in hormonal therapy resistance.

Critical knowledge gaps persist regarding CHAC1’s role in urothelial carcinomas (e.g., bladder cancer), where mechanistic investigations remain absent. Additionally, stage-specific regulatory networks in ccRCC and CHAC1-androgen receptor crosstalk in prostate cancer warrant deeper mechanistic dissection.

Translational perspectives emphasize CHAC1’s clinical utility, encompassing its application as a dynamic biomarker for ccRCC risk stratification and disease staging, the development of CHAC1-targeted therapies including activators and microenvironment-modulating agents (e.g., exosomal miRNA inhibitors) to reverse chemoresistance, and rational combination strategies with androgen pathway inhibitors or immune checkpoint modulators. Translating these approaches through rigorous preclinical validation and clinical trials offers significant potential to overcome therapeutic resistance in urological malignancies.
